# Hypertension in living kidney donors: understanding the real risk

**DOI:** 10.1093/ckj/sfae244

**Published:** 2024-08-13

**Authors:** Gizem Kumru, Krista L Lentine

**Affiliations:** Department of Nephrology, Ankara University School of Medicine, Ankara, Turkey; Saint Louis University Center for Abdominal Transplantation, SSM Health Saint Louis University Hospital, St. Louis, MO, USA

Increasing use of organs from living donors helps bridge the gap between the need for and supply of donated organs and offers patients with end-stage kidney disease the best opportunity for long-term, dialysis-free survival [1]. While the benefits of living donor kidney transplantation (LDKT) for recipients are well established, the possible risks for living kidney donors (LKD) must be accurately quantified and disclosed to support potential donors and families in fully informed consent and shared decision-making. Reductions in glomerular filtration rate (GFR) are associated with higher blood pressure (BP) in disease states and animal models, raising plausible concerns for increased risk of hypertension after donor nephrectomy. Various studies have evaluated the long-term risk of hypertension after living kidney donation, with many finding that there may be an increased risk compared to healthy nondonor controls (Table 1). However, uncertainty has remained due to limitations of study design, follow-up, and potential bias with available controls.

**Table 1:** Summary of key studies examining hypertension and change in BP in LKD.

Author, year	Study design/population	Follow-up	Key findings
Boudville *et al*. 2006 [5]	Meta-analysis of 48 studies from 28 countries that enrolled 5145 LKDs compared with control participants	At least 5 years	Higher mean systolic (weighted 6 mm Hg, 95% CI 2–11 mm Hg), and diastolic (weighted 4 mm Hg, 95% CI 1–7 mm Hg) BP in LKDs
Garg *et al*. 2008 [6]	Retrospective of 1278 LKDs in Ontario, Canada between 1993 and 2005 compared with 6359 healthy adults	Mean 6.2 years	Hypertension more common in LKDs compared with nondonors (16.3% vs 11.9%; HR, 1.4; *P *< .001)
Lentine *et al*. 2010 [7]	Retrospective study of 4650 US LKDs between 1987 and 2007	Median 7.7 years	Prevalence of new-diagnosed hypertension after donation was 17.8% (95% CI 15.8–20.2) at 5 years with higher adjusted risk among Black (aHR 1.52; *P *< .001) and Hispanic (aHR 1.36; *P *< .001) compared with White LKDs
Sanchez *et al*. 2018 [8]	Longitudinal study of 3700 LKDs between 1963 and 2014 at Minnesota, USA	Mean 16.6 years	Hypertension incidence at 5, 10, and 40 years 4%, 10%, and 51%, respectively, and was associated with adverse events including, proteinuria; eGFR < 60 ml/min/1.73 m^2^, cardiovascular disease, and death
Holscher *et al*. 2019 [9]	Longitudinal, US multicenter study of 1295 LKDs compared with a weighted cohort of 8233 healthy nondonors from two national cohort studies	Median 6 years for donors and median 23 years for controls	19% higher risk of self-reported hypertension associated with kidney donation (aHR, 1.19; *P *= .04), which did not vary by race
Kasiske *et al*. 2020 [10]	Prospective cohort study of 205 LKDs and 203 paired controls	9 years	No difference in BP determined by 24-hour monitoring or in incidence of hypertension between donors and controls
Krishnan *et al*. 2020 [11]	9750 LKDs from UK Transplant Registry between 2001 and 2013 compared with 19 071 healthy donors	Median 8.4 years for donors and median 5.5 years for controls	Higher risk of hypertension LKDs at 5 years (OR, 0.66; *P *< .001) but not at 10 years (OR, 0.86; *P *= .048)
Chaudry *et al*. 2020 [12]	Retrospective study of 1262 Danish LKDs between 1996 and 2017 compared with age- and sex-matched 12 620 controls	Median 7 years	Higher 10-year absolute risk of hypertension LKDs compared with controls (4.2% vs 2.4%; absolute risk ratio, 1.64; *P *< .05)

OR, odds ratio.

On this backdrop, a new study by Garg *et al*. examines a prospective, multicenter cohort study to evaluate the risk of hypertension in LKDs and nondonors [2]. The study compared 924 “standard criteria” LKDs to 396 healthy nondonors in Canada and Australia. Standard criteria donor was defined as: age between 18 and 70 years, body mass index (BMI) <35 kg/m^2^, normal BP at donation (systolic and diastolic BP < 140/90 mmHg), normal serum creatinine level (<115 μmol/l in men or <90 μmol/l in women), and absence of comorbidities that would contraindicate donation. A larger “pseudosample” of 928 nondonors was produced by inverse probability of treatment weighting on a propensity score to balance nondonors with donors on baseline characteristics. The participants completed blood and urine laboratory testing and at-home BP measurements annually during a median postdonation follow-up period of 7.3 years. During follow-up, the risks of hypertension {17% vs 17%; weighted hazard ratio [HR], 1.11 [95% confidence interval (CI), 0.75–1.66]} and albuminuria [15% vs 11%; weighted HR, 1.46 (95% CI, 0.97–2.21)] were not significantly different between the donors and nondonors. The longitudinal change in mean BP, major cardiovascular events and death were also similar. Although the mean annual estimated GFR (eGFR) decline was slower in donors compared with nondonors (0.1 vs −1.2 ml/min/1.73 m^2^ per year, respectively), the final median eGFR was lower in donors due to the initial drop after donor nephrectomy (mean 32 ml/min/1.73 m^2^). No participants developed end-stage kidney disease, but an eGFR between 30 and 60 ml/min/1.73 m^2^ at least once in follow-up was more common in donors than nondonors (47% vs 5%).

This well conducted study provides important data supporting minimal impact of living kidney donation on hypertension risk in “standard donors.” However, there are important considerations in applying this research to practice. The study population was predominately White (88%), limiting generalizability to other racial and ethnic groups. Racial disparities in the risks of postdonation hypertension have been demonstrated for Black and Hispanic LKDs [3]. Increased risk of hypertension has been demonstrated for Black donors not only compared to White donors but also compared to race-matched healthy nondonors [4]. Therefore, the results of the study may lead to a reduced appreciation of donation-attributable risk in the non-White population. The convenience sample of nondonors was less than half the size of the donor sample and confidence intervals for some of the outcomes were wide and imprecise. While propensity score-based methods were used to create a larger pseduosample, results may differ from comparison to larger samples of nondonors. Donor candidates with medical complexity outside the standard criteria used in this study may be accepted for donation in contemporary clinical practice. Garg *et al*. also found that eGFR <60 ml/min/1.73 m^2^ and albuminuria occurred more often in these standard donors than nondonors. It is also possible that additional risks may emerge with longer follow-up. Thus, the findings also underscore the importance of lifelong follow-up of donors, especially for these outcomes that may be associated with other health complications.

We applaud Garg *et al*. for advancing the quality of evidence available for donor counseling. Based on these new data, “standard” White-race living donor candidates can be counseled about the relative safety of living kidney donation for hypertension risk. However, risk is not “one-size fits all,” and tailoring to individual demographics and health profiles is critical, along with ongoing efforts to strengthen the evidence for non-White donors. Thus, we encourage ongoing studies investigating the long-term impact of LKD on hypertension risk according to race and other baseline health factors, as well as the impact of postdonation hypertension on downstream clinical outcomes including kidney disease and cardiovascular health.
